# Interdisciplinary Animal Research Ethics—Challenges, Opportunities, and Perspectives

**DOI:** 10.3390/ani14192896

**Published:** 2024-10-08

**Authors:** Marcel Mertz, Tatiana Hetzel, Karla Alex, Katharina Braun, Samuel Camenzind, Rita Dodaro, Svea Jörgensen, Erich Linder, Sara Capas-Peneda, Eva Ingeborg Reihs, Vini Tiwari, Zorana Todorović, Hannes Kahrass, Felicitas Selter

**Affiliations:** 1 Institute for Ethics, History and Philosophy of Medicine, Hannover Medical School, 30625 Hannover, Germany; thetzel@proton.me (T.H.); kahrass.hannes@mh-hannover.de (H.K.); selter.felicitas@mh-hannover.de (F.S.); 2 Section Translational Medical Ethics, National Center for Tumor Diseases (NCT) Heidelberg, Department of Medical Oncology, Heidelberg University Hospital, Medical Faculty Heidelberg, Heidelberg University, 69120 Heidelberg, Germany; karla.alex@med.uni-heidelberg.de; 3 Department of Law, Freie Universität Berlin, 14195 Berlin, Germany; katharina.braun@fu-berlin.de; 4 Department of Philosophy, University of Vienna, 1010 Vienna, Austria; samuel.camenzind@univie.ac.at; 5 Department of Philosophy and Humanities, Freie Universität Berlin, 14195 Berlin, Germany; rita.dodaro7@gmail.com; 6 Humanities Department, Università della Calabria, 87036 Rende, CS, Italy; 7 Department of Applied Animal Science and Welfare, Swedish University of Agricultural Sciences, 750 07 Uppsala, Sweden; svea.jorgensen@slu.se; 8 Messerli Research Institute, University of Veterinary Medicine, 1210 Vienna, Austria; erich.linder@vetmeduni.ac.at; 9 Vienna Doctoral School of Philosophy, Department of Philosophy, University of Vienna, 1010 Vienna, Austria; 10 i3S–Instituto de Investigação e Inovação em Saúde, Universidade do Porto, 4200-135 Porto, Portugal; sara.capas@i3s.up.pt; 11 ICBAS School of Medicine and Biomedical Sciences, University of Porto, 4050-313 Porto, Portugal; 12 Karl Chiari Lab for Orthopaedic Biology, Department of Orthopedics and Trauma Surgery, Medical University of Vienna, 1090 Vienna, Austria; eva.reihs@meduniwien.ac.at; 13 Institute of Neuronal Cell Biology, Technical University Munich, 81377 Munich, Germany; v.tiwari@tum.de; 14 German Center for Neurodegenerative Diseases, 81377 Munich, Germany; 15 Graduate School of Systemic Neurosciences, Ludwig-Maximilians-Universität, 82152 Planegg, Germany; 16 Department of Philosophy, University of Belgrade, 11000 Belgrade, Serbia; zoranatod@gmail.com; 17 Center for the Study of Bioethics, 11000 Belgrade, Serbia

**Keywords:** animal experimentation, animal ethics, animal law, animal ethics committees, harm–benefit analysis, 3Rs

## Abstract

Can nonhuman animals be used for the benefit of humans in a scientifically and morally justified manner and, if yes, how? Based on our own experiences as scholars from various academic backgrounds, we argue that this question can only be answered as an interdisciplinary and international endeavor, considering insights from research ethics and animal ethics as well as scientific and legal aspects. The aim of this article is to contribute to the foundation of the emerging field of animal research ethics. In doing so, we describe the following seven phases of animal research experiments: ethical, legal and social presumptions (phase 0), planning (phase I), review (phase II), conduct of experiments (phase III), publication/dissemination (phase IV), further exploitation of results (phase V), and evaluation (phase VI). In total, 16 key ethical, legal, and practical challenges that an ethical framework for the use of animals in research needs to address are identified and analyzed. Finally, we characterize the following four meta-challenges and opportunities associated with animal research ethics as a field: (1) moral pluralism, (2) the integration of views and positions outside the laboratory, (3) international plurality of conduct, standards, and legal norms, and (4) interdisciplinary education.

## Simple Summary

Are we morally justified in using animals in biomedical research and if so, how can we make sure that the experiments are conducted in a scientifically and morally acceptable manner? Based on our own experiences as scholars from various academic backgrounds, we argue that this question can only be answered as an interdisciplinary and international endeavor. Thus, our article aims to contribute to the foundation of the emerging field of animal research ethics, combining perspectives from research ethics, animal ethics, science, and law. In doing so, we describe the following seven phases that animal experiments typically run through: ethical, legal and social presumptions (phase 0), planning (phase I), review (phase II), conduct of experiments (phase III), publication/dissemination (phase IV), further exploitation of results (phase V), and evaluation (phase VI). Here, 16 key ethical, legal, and practical challenges are identified and analyzed that need to be addressed. Apart from challenges arising at the level of the experiments themselves, there are also so-called meta-challenges associated with animal research ethics as a field. Four of these are presented and further discussed, also in relation to their opportunities for the further development of animal research ethics that takes into account interdisciplinary and international perspectives.

## 1. Introduction

In the context of using nonhuman animals (hereinafter referred to as “animals”) in scientific experimentation, many ethical conflicts arise. In this article, we primarily focus on research where animals are used as models for humans in the course of biomedical research. We acknowledge that a large number of animals is also used in research for other purposes, some of which are intended to benefit animals (studying animal diseases, investigating animal sentience), others are intended to benefit humans but are not required for human survival (such as animal use for cosmetics testing). In our view, one of the most relevant questions in animal research is as follows: (How) may animals be used for the benefit of humans in a manner that is both scientifically and morally justified? (It should be noted that the question, “Can animals be used for the benefit of humans in a manner that is both scientifically and morally justified?” without the “How” may be the focal question for those who take either an “anything goes” perspective [1] or an abolitionist position that rejects all use of animals in research [2,3,4]).

We think that this question is all the more pressing in the process of designing and testing novel hypotheses in biomedical research, which aims to generate knowledge for therapeutic purposes and thus ultimately for the benefit of humans. Thoroughly examining the conditions under which such research involving animals may or may not be permissible requires a robust ethical framework. The development and implementation of such a framework faces the challenge that the ethics of animal experimentation in biomedicine (hereinafter referred to as “animal research”) involves diverse perspectives, multiple scientific and non-scientific academic disciplines, and interests. If this task should be approached in collaboration, it thus needs to reconcile sometimes conflicting positions, as researchers, veterinarians, and animal ethicists, for example, may hold differing moral views.

Such a shared ethical foundation has previously been discussed under the term animal research ethics [5,6,7]. However, animal research ethics is still a developing field. In our view, the goal of animal research ethics at this moment in time should be to develop an ethical framework for the use of animals in research while considering research ethics, animal ethics, scientific aspects, as well as the associated legal requirements and international differences. The aim of this article is to contribute to the foundation of the emerging field of (interdisciplinary and international) animal research ethics. The endeavor of developing a “universal” animal research ethics is an optimistic idea we are striving towards but which is certainly challenging to realize in full (see also Limitations section). By “interdisciplinarity”, in this context, we mean not all the scientific disciplines involved in animal research but primarily a cooperation between the “ELSI (=ethical, legal, and social issues)” disciplines (represented by ethics, law, social sciences, and potentially other humanities) and the sciences (represented by the multiple disciplines experimenting with animals). The chosen approach is a description of what are—based on our own experiences as scholars from various disciplines and informed by the previous literature—some of the key challenges throughout the different phases of an animal experiment and what are the opportunities associated with the ethics of animal experimentation.

**Figure 1:**
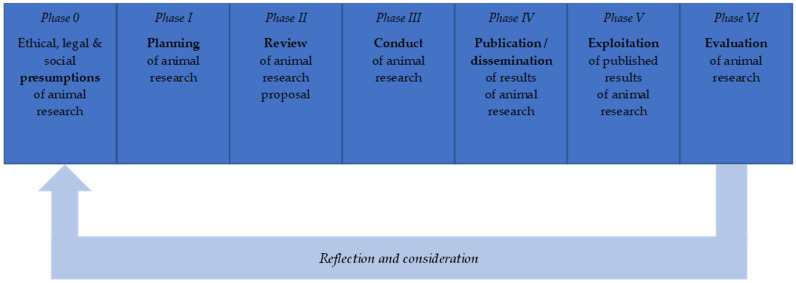
Seven phases of animal research (viewed from animal research ethics).

Following a succinct summary of our methodology (Section 2), we start Section 3 (Results) with a brief overview of the regulatory issues associated with animal research in general (Section 3.1). Thereafter, we offer an account of seven phases of animal research as cornerstones for an (interdisciplinary) animal research ethics (Section 3.2). The seven phases are as follows: ethical, legal and social presumptions (phase 0), planning (phase I), review (phase II), conduct of experiments (phase III), publication/dissemination (phase IV), further exploitation of results (phase V), and retrospective evaluation (phase IV) (Figure 1). This is followed by an analysis of the key ethical, legal, and practical challenges of each of the seven phases, where we identify a total of 16 challenges that are summarized in Table 1 (Section 3.3, Section 3.4, Section 3.5, Section 3.6, Section 3.7, Section 3.8 and Section 3.9), as well as some overarching further challenges (Section 3.10). Thereafter, we describe the meta-level challenges as well as opportunities associated with establishing animal research ethics as a field (Section 3.11). These challenges are separate from the phases themselves and instead pertain to questions of “how” to study, discuss, and advance in respect of the challenges associated with the seven phases of animal research.

The article is based on joint work of participants of the retreat week “Animal Research Ethics as Interaction of Research Ethics, Animal Ethics and (Animal Protection) Law: International Perspectives on Theoretical and Cultural Differences”, which took place from 5 to 9 September 2022 in Hannover, Germany, and was funded by the German Ministry for Education and Research (BMBF). During the retreat week, participants and invited speakers from different disciplines (e.g., life sciences, veterinary medicine, philosophy/ethics, law) explored the ethical and regulatory challenges related to (increasingly) international animal research ethics and discussed the conception of this emerging field (for a conference report, see [8]; see also the poster which emerged from this retreat [9]).

The multi- and interdisciplinary perspectives of the contributors shaped the selection, weighing and presentation of the ethical challenges, opportunities, and perspectives associated with animal research ethics outlined in this article. Diverse perspectives from the different disciplines are visible and mutually informative. This inclusive approach ensures an in-depth exploration of the given challenges without being limited to a particular perspective (legal, ethical, scientific, societal). While it is not the aim of this article to give an exhaustive summary of all issues currently discussed in animal research ethics or animal ethics or even provide a complete account of an international animal research ethics, this approach addresses a wide array of issues, laying the foundation for further studies. It points towards the variety of questions and challenges of animal research ethics to enhance further interdisciplinary discourse.

## 2. Materials and Methods

At the end of the retreat week, the basic orientation of the presentation and structuring was jointly decided, primarily on a phase model of animal research (see Figure 1). The organization team of the retreat week (M.M., H.K., F.S., T.H.) then created a rough structure of the manuscript and formulated questions for individual areas, especially for the various challenges, to the co-authors (the participants of the retreat week). These questions were answered individually by each author and sent back to the organization team, who also took on the role of an editor here. The “editors” reviewed all the contributions and sensibly added text segments from them to the intended sections of the manuscript. This first version of the manuscript was then circulated several times among all authors, who suggested and incorporated changes in the outline, structure, and content, until a final version that could be consented to by all the authors could be reached.

## 3. Results

### 3.1. Some Regulatory Challenges Associated with Animal Research

The regulatory landscape pertaining to animal research reflects some of the underlying ethical and scientific tensions. Directive 2010/63/EU [10] of the European Parliament and of the Council of 22 September 2010 on the protection of animals used for scientific purposes serves as an example of this nexus—as much as the legal and regulatory matters are addressed, this article primarily focuses on European Union (EU) law where the primary expertise of the authors lies. The Directive proclaims the need for animal protection given their “intrinsic value” (preambulatory clause 12) and “ability to experience pain, suffering, distress and lasting harm” (preambulatory clause 8), while also maintaining that “the use of live animals continues to be necessary to protect human and animal health and the environment” (preambulatory clause 10). Consequently, the Directive also makes distinctions based on animal species. It explicitly limits its application to vertebrates and cephalopods (Art. 1 (3)). Further, primates are afforded additional protection (Art. 8), inter alia due to their “genetic proximity to human beings” (preambulatory clause 17). While similar tendencies might be found in various jurisdictions (for an overview and comparison, see [11]), the regulatory framework should not be confused with trends in the practice of animal research. For example, despite the widely acknowledged need for special protection for primates, the use of primates might be declining in the European Union (EU) but is increasing in non-EU jurisdictions [12].

Looking at the regulatory landscape and the scientific practices from an international perspective is essential for the emerging field of animal research ethics, given that scientific research is oftentimes conducted within international consortia and corporations. Different legal frameworks might, in some cases, limit international collaboration; in other cases, they might encourage researchers from countries with stricter regulations to move to a jurisdiction that allows them to conduct certain animal research. Based on the understanding that regulations are shaped by ethical views, the latter has also been called “ethics dumping” [12]. Thus, the international dissemination of animal research is ethically challenging due to the fact that there are differing laws and regulatory practices in place across the world.

Ethical and regulatory challenges are further complicated by new scientific developments. For example, genetic modification allows us to create animals that have specific characteristics relevant for the investigation of a respective disease or possible treatments; the use of genetically engineered mice is a major pillar in current biomedical research [13,14,15]. But from a scientific perspective at least, the translation of genetic engineering from animal research to humans also requires the testing of these technologies on other mammals, including primates, which in turn comes with additional ethical and regulatory challenges [16,17].

Summing up, as an international discipline, animal research ethics faces a profound tension between, on the one hand, existing law and practices regarding the use of animals in research and, on the other hand, ethical demands that cannot be met within these parameters.

### 3.2. Phases of Animal Research and Associated Ethical and Legal Presumptions

One of the key challenges for (international) animal research ethics is to find a constructive way to deal with (a) competing legal positions, (b) competing ethical positions, ranging from an “anything goes” view ([1], 246 f.) to an ambitious abolitionist position that rejects all use of animals in research, e.g., based on animal rights [2,3,4], and (c) the status quo of animal use for research purposes. For this, a robust animal research ethics requires a systematic analysis of challenges resulting from the differences in the status quo and sometimes much more ambitious ethical demands. The presentation of the ethical, regulatory and, to some extent, practical challenges of animal research is based on a seven-phase model (see Figure 1). This phase model is a simplified representation of the process of animal research. However, it recognizes that it is possible to move back and forth between the different phases, even if there is a clear direction from left to right.

Further, existing phase models have been taken into account. While the phase model of animal research by Röcklinsberg and colleagues [18] describes the potential misconduct or questionable research practices of animal researchers in four phases, with our seven-phase model, we provide a more fine-grained approach with an emphasis on the activities and (pre-)conditions present from the beginning to the end of an animal research project. Our model is, however, still an abstraction and idealization, to a great extent, of the activities that are often intertwined in reality. Moreover, animal experiments are typically accompanied by a range of different (non-animal) methods as well (such as in vitro or in silico methods). In the following, the seven phases are briefly described and thereafter discussed in more detail with respect to the selected specific challenges and opportunities associated with each phase of animal research in Section 3.3, Section 3.4, Section 3.5, Section 3.6, Section 3.7, Section 3.8 and Section 3.9.

Phase 0 encompasses presumptions of animal research. These include preliminary assumptions or positioning, especially of an ethical nature, but also the existing legal framework and social norms. The name “Phase 0” expresses the fact that these are not research activities as such. However, since these preliminary assumptions inform the subsequent phases, they cannot be omitted. Phase 0 permeates the subsequent phases, because they constitute the prerequisites and premises that the current practice of animal research is based on.

Phase I describes the planning of animal research. This includes everything from goal setting, design, review of existing knowledge, search for alternative methods, preparation of the application for the project evaluation—also colloquially referred to as an ‘ethical review’—to securing the financial and infrastructural measures that will be required for a research project (if approved).

Phase II describes the process and (then) the results of the project evaluation of an animal research proposal. In many countries, including all the EU member states, it is a mandatory procedure to seek ethical approval for conducting research on live vertebrates (and occasionally other animals) [19]. The aim of this step is to ensure ethical evaluation as well as adherence to internationally set standards (e.g., [10]) and national law. In the EU, determining if a project should be granted ethical approval or not falls upon national “competent authorities” commonly referred to as Animal Ethics Committees (AECs) (see Directive 2010/63/EU Article 3 p. 7 for definition of the term and Section 3 Article 36 and 38 for descriptions of the task of the competent authority).

Phase III concerns the actual performance of the research itself, which is why aspects of overview of the handling of research materials, including the research animals, are also covered. The conduct (potentially also including the preregistration of the study) follows international standards and, again, mainly national legislation.

Phase IV concerns the publishing and dissemination of results once the data have been generated, e.g., via conference contributions, scientific papers, or lectures.

Phase V encompasses the further exploitation of the results of the research, e.g., in the context of translational research. The development of new projects (new questions, new hypotheses) based on the results can also be seen as part of this phase. This phase can therefore also take place in parallel with phase IV and might likely take place in parallel with phase VI.

Phase VI describes evaluation and further (more long-term oriented) interpretation of research results. “Evaluation” at this stage is not limited to the retrospective evaluation (in line with both international and national standards (see Art. 39 in [10]) but can also encompass broader ethical and scientific aspects of the results or methods.

Each of the seven suggested phases of animal research comes with specific ethical, legal, or practical challenges. These challenges are not only faced by those reviewing the research from an ethical perspective (in phase II), but by all those involved in the different phases of animal research. Key challenges of animal research associated with each of the seven phases of animal research are outlined in what follows (for a summary, see Table 1).

### 3.3. Challenges of Phase 0: Ethical, Legal, and Social Presumptions of Animal Research

The challenges of phase 0 primarily arise from the normative and legal presumptions of animal research. These presumptions are intertwined with the various stages of animal experimentation and with the meta-theoretical considerations of animal research ethics (see also Section 3.11). The four challenges associated with phase 0 we focus on are as follows: “Normative pluralism”, “Anthropocentrism and the perspective of animals”, “Operationalization of ethical principles”, and “Animal rights and consent”.

#### 3.3.1. Challenge 1: Normative Pluralism

The regulatory landscape and practices of animal research build on different ethical background assumptions. The absence of a stringent code of conduct also raises questions about responsibility and accountability for actions undertaken towards animals, including who should be held accountable and to what extent.

The challenge of normative pluralism furthermore emerges from the interdisciplinary ambitions of animal research ethics (that comes with its own set of challenges which will be further explored in Section 3.11). Animal research ethics needs to consider the norms from various fields, e.g., scientific, economic, legal, and ethical. For example, using a higher number of animals for an experiment might increase the scientific quality of an experiment but bring about more animal ethical concerns. In addition to the challenging ethical and scientific trade-offs, there are cases of incommensurability within the moral norms themselves. That is, cases in which two moral “oughts” require to engage in incompatible courses of action [20].

Consequently, a key challenge of phase 0 is the search and foundation of a common normative position. This includes questions about how the interests or status of animals should be considered morally, what their moral significance is, and how they relate to other morally relevant goods such as scientific freedom or human health.

#### 3.3.2. Challenge 2: Anthropocentrism and the Perspective of Animals

One could argue that with respect to conducting animal research, there is a tendency to regard animals as objects of research that might have value in themselves but primarily have instrumental value to be used by humans as they see fit. This is an anthropocentric position placing humans (ánthropos = human being) at the center of moral consideration. Anthropocentrism is in stark contrast to a broad consensus among animal ethicists that sentient animals have interests, which, in the context of research, includes the interest in not suffering or in not being harmed and the interest in continued life or in not being killed by experimentation [21,22,23,24,25,26].

From the perspective of animal ethics, one problem is that the anthropocentric justification of animal research is grounded in metaphysical premises (e.g., that humans are the “pride of creation”) and facts about the world (e.g., that humans and animals are categorically different beings). Some argue that such premises and assumed facts have been refuted by natural sciences (e.g., by evolutionary biology and neurosciences) and their often-associated naturalistic world view [27,28]. A related problem is that the implicit anthropocentric premises animal research is built on are assumed to be self-evident and therefore are rarely considered in need of justification.

#### 3.3.3. Challenge 3: Operationalization of Ethical Principles in Legal Norms

The challenge to operationalize ethical principles arises where the goal is to develop a common normative position for an international and interdisciplinary animal research ethics. This challenge can be illustrated with the following two examples:

(1) Following the Declaration of Helsinki from the World Medical Association for human subject research [29], according to Petkov et al. [30], an amended version of the 2010 Basel Declaration (“A call for more trust, transparency and communication on animal research”) for animal research [31] should serve as a more complete animal research declaration, which includes a unified set of principles. However, the Basel Declaration still gives human interests a lexicographic priority over animal interests [27]. But this human priority paradigm might not be universally ethically acceptable (see challenge 2).
(2) It is open to debate, if or to what degree the famous 3Rs (Replace, Reduce, Refine) [32] which are underlying the Directive 2010/63/EU [10] are meant to protect animals or “just” improve scientific results, as Camenzind and Eggel [33] have argued. If it were only a matter of improving scientific work, it would be questionable whether the ethical principles are operationalized into legal norms through 3R principles and whether legal protective effects only “coincidentally” follow from the 3R principles.

The assumption (or the traditional view) that animals do not have moral status or have a lower moral status than humans is also reflected in their legal status. Even though it has been recognized by the Lisbon Treaty, as well as the civil codes and animal welfare laws of many European countries, that animals are not things but sentient beings, they may still be treated as things and often are subject to laws that apply to things [34,35]. This legal situation of animals can have an impact on how they are treated in research, and how well they are protected by the legal regulations relating to research.

#### 3.3.4. Challenge 4: Animal Rights and Consent

Another ethical challenge arises from the sheer incapacity of animals to give consent. As Richard Healey and Angie Pepper [36] have explained, animals lack the capacities that are necessary to form the complex intentions required to waive a right by giving consent. Another central issue is that consent in the context of human research must be informed, which would require, in the context of animal research, that animals understand why participation in research activities is asked from them by humans, but this is not possible because of a language barrier [37]. This comes with the problem of potential exploitation of animals as (non-consenting) research subjects, which would be prima facie unacceptable for humans. It also comes with the practical challenge of determining the potential interests of animals in research. Since animals cannot speak for their interests themselves, humans need to assess whether any particular study that involves animals justifies the harms inflicted on them. Against this backdrop, animals’ incapacity to consent should also be recognized as a legal issue.

To fill the void left by the inapplicability of consent, Angela Martin suggested that a consent-related notion such as assent and dissent (expression of a will without legal capacity to give consent, originating from medical ethical discussions on children’s inability to consent) can be used in the context of research involving animals [38]. Assent would not be a standalone criterion according to Martin but come alongside other criteria ensuring overall that animal rights are respected in animal research. Similarly, Holly Kantin and David Wendler [39] suggested that respecting an animals’ dissent or even soliciting their assent may be ethically required, first and foremost, for animal welfare reasons but, in the cases of some animals such as chimpanzees, also to respect animal agency.

Despite the potential of these proposals to improve animal welfare, they leave crucial questions unanswered. Assent/dissent models cannot provide the overarching legitimizing framework for research involving animals, as they do not shed sufficient light on the context in which research takes place (see also phase VI) and, in particular, on the power imbalances between human researchers and animals. Furthermore, it remains open what an animal assent or dissent could look like in practice.

### 3.4. Challenges of Phase I: Planning of Animal Research

The planning phase of animal research encompasses many different aspects from other phases as well, especially from phase 0. It is, however, clearly distinguishable since this phase focuses on challenges surrounding the process of proposal writing and concrete preparation (e.g., to create infrastructure) for a specific research project. The following two issues seem especially important here: “Comparability to human conditions when using animal models in medicine” and “Refraining from planning animal research–the search for alternatives”.

#### 3.4.1. Challenge 5: Comparability to Human Conditions When Using Animal Models in Medicine

If the decision to conduct animal research is made in phase 0, there are various challenges in choosing the most fitting animal model. One major challenge concerns consideration about the degree of comparability to human conditions using an animal model. The problem is reflected by the difficulties that can occur when trying to define a scientifically accepted and generally valid consensus about the number and exact categorization of the major observable types of a human condition that may have a different trigger but the same final pathophysiological consequence in animals [40]. The uncertainty on how a disease manifests as the resulting condition can lead to difficulties when planning for disease recapitulation attempts in an animal model [41]. Since similar problems exist when human diseases are modeled without the use of animals (we thank an anonymous reviewer for pointing this out), the question arises on whether using animals is preferable to non-animal models with respect to how fitting the model is for the (human) disease. Moreover, it can be argued that the issue of comparability is especially pressing in the case of animal models, since the comparability to human conditions relates to the justification of using sentient animals in potentially harmful or painful ways.

All in all, it is the experimenters’ responsibility to use an animal model fit for the study that is conducted. This also implies open-minded investigators able to question themselves and their proceeding in terms of the eligibility of the chosen methods. Paradigm changes in science require an adaptation of the applied experimental strategies and elimination of outdated methods based on tradition.

#### 3.4.2. Challenge 6: Refraining from Planning Animal Research—The Search for Alternatives

**Figure 2:**
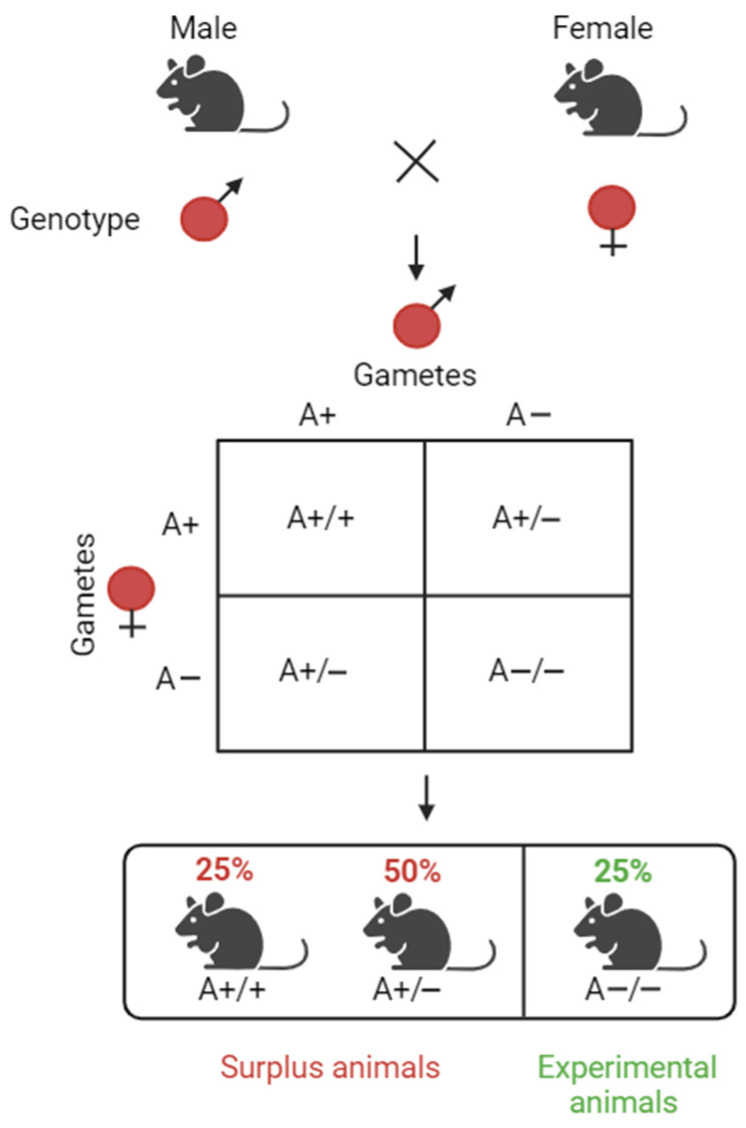
Natural breeding laws out of control of researchers and the problem of surplus animals.

For scientists, it is essential to design experiments with an adequate number of animals and suitable statistical analyses to achieve a scientific goal that improves the reliability of the methods and validity of the results. When striving to safeguard the methods and results, it is important to keep in mind, however, that for each laboratory animal calculated, many times more animals must be produced. In 2022, 9,556,700 animals were bred, killed and not used in the EU, including those killed for organs/tissues as well as for health reasons, either of the individual animal or the colony. A large majority of these animals, namely 5.9 million, were bred and killed for the maintenance of an established genetically altered line [42]. This can be exemplified by the breeding process of genetically modified animals, for example, mice, during which animals without the required traits are unavoidably also born. If a researcher wishes to study the effect of a knockout gene (A-/-), only 25% of the animals can be used as experimental animals, while the remaining 75% may be used partially in animal experiments (e.g., as control animals or for further breeding). This is the result of natural breeding laws, over which the researcher has no control, see Figure 2 [43].

This shows that the goal of reducing the number of animals used in research conflicts with the goal of refinement (from the 3Rs) and must be carefully balanced. Furthermore, a reduction in animals could mean that the remaining animals are subject to more procedures and more suffering. While this dilemma cannot be resolved, regulation may strike a balance. Importantly, Article 16 of Directive 2010/63/EU stipulates that animals involved in procedures classified as ‘severe’ are not to be reused (we thank an anonymous reviewer for pointing out this aspect), apart from in exceptional circumstances—if the conditions in Article 16 (2) are met—whereby even then, the animal may not be reused in a second procedure classified as ‘severe’.

In this respect, more attention should also be paid to prevent confusion between reducing animal experimentation and replacing or avoiding animal experimentation since these goals and the means to achieve them differ. Improving the quality of animal experiments can reduce both the number of animals used and the total number of experiments (by avoiding redundant experiments, for example). This does not mean that an alternative method is chosen to replace an animal experiment.

The situation is further complicated by the question of sentience and cognitive abilities of animals used in research. The knowledge of these attributes is rapidly evolving and has the potential to bring about major changes in the ethical use of animals in research. Since more species are now considered sentient [44], various fields will need to address this and provide clear ethical guidelines on the use of species that, until now, were considered non-sentient and consequently not legally protected.

Even if the attempt is made to plan research with alternative, “animal-free” methods and the issue of “surplus” animals can thus be prevented, one should be aware of the often-existing dependence on animals to achieve certain results. Doing research without an animal experiment does not necessarily mean doing animal-free research, as the use of animals in science is not only limited to those who do hands-on experiments with them. Lab scientists are strongly dependent on many animal-derived materials to keep running the simplest experiments and to analyze the outcome [45]. In daily lab life, scientists probably often simply tune out the sometimes problematic circumstances of the production pipeline that are undeniably an ethical challenge. Even though there is increased public attention to the use of animals in research and the resulting demand to phase out their inclusion, a comparable trend is hardly seen when it comes to animal-derived lab supply. An additional issue is that many scientists might not be aware of the replacements for the animal-derived products regularly used in the lab that already exist; consequently, the degree of a recognizable change is still limited. Another problem is a low incentive to swap the standard because every small modification on the used and defined standards (e.g., reagents and materials) would logically require the recalibration of the whole model to either outdate any unwanted influence on the result or to set a new verification baseline.

### 3.5. Challenges of Phase II: Ethical Review of Animal Research Proposals

This phase encompasses challenges that both the scientists submitting the animal research proposal and the members of the Animal Ethics Committee (hereinafter referred to as “AEC”) assessing the research proposal face (such committees can also have different names; UK: Animal Welfare and Ethical Review Body (AWERB) [46]; EU: Animal Ethics Committees (AECs) [10]; USA: Institutional Animal Care and Use Committees (IACUC) [47,48]. Challenges surrounding the review of animal research proposals are manifold and many of the ones often discussed concern the harm–benefit analysis (HBA), understood both as a tool and a process whereby the expected “suffering, pain and distress” to the animals (colloquial harm) is weighed against the “expected outcome” of the project (colloquial benefits) (Art. 38 p.2d [10]). Only if harm and benefit balance out may a project receive ethical approval.

Several scientific studies have, during the past few years, shed light on the difficulties within and surrounding the project evaluation or ethical review process of animal research [49,50,51,52,53]. This focus is important because the project evaluation ultimately determines which animal research is conducted within the EU, the details of said animal use, and its scientific deliverables. Examples of areas of concern have been and still are the following: the composition, competence and internal discussion climate of the AECs tasked with performing the ethical review [49,50,51,52,53]; the limitations of HBA as the appointed method for ethical review [49,54,55,56,57]; inadequate application of replacement, reduction, and refinement (3Rs) in planning (see phase I as discussed above) and in reviewing research projects [49,53,58,59]; a lack of transparency and public insight into the use of animals for research purposes and into the review process in particular [60]. Although all the above concerns are important, we argue that the most pressing issue to deal with is that the tool provided—and, e.g., in the EU, is legally required to be used—for the ethical review, the HBA is, in fact, alarmingly often unfit for its intended purpose [56,61]. A direct consequence of this is that, despite the weighing of positives against negatives appearing rather straightforward, the ethical review is far from easy when performed in practice.

Following this brief literature overview on the challenges associated with HBA, we now outline the following three selected challenges associated with phase II: “Insufficient considerations for animal interests in the tools of ethical deliberation”, “Measuring the unmeasurable”, and “Comparing apples with oranges: animal harms vs. human benefits”.

#### 3.5.1. Challenge 7: Insufficient Consideration for Animal Interests in the Tools of Ethical Deliberation

In our experience, in the HBA, different weights are assigned to the interests and moral rights of animals versus those of humans. Non-human animals do not have the same legal status as humans, and their interests are placed on a scale likely tipped in favor of the interests of human society [62]. As such, despite the utilitarian influences of Directive 2010/63/EU [10] visible in the HBA in Europe, well-known utilitarian approaches such as Bentham’s dictum “everybody to count for one, nobody for more than one” or Singer’s “principle of equal consideration of interests” [63] are potentially not always adhered to within the ethical review process. It can also be argued that the HBA implicitly relies on a particular way of looking at and conceptualizing animals, i.e., as research tools or utility vessels [64]. This way of conceptualizing animals—although it allows us, for example, to talk about them within a utilitarianism-oriented framework—will likely preclude the consideration of the discourses about rights and justice that would, on the other hand, be available if we would talk about them as beings with inherent values [3].

According to Hills, within the HBA, animals are not seen as worthy of equal consideration compared to humans, and one could argue that this makes the weighing of animal interests against human interests not only difficult but inherently biased [65].

The AECs, being the appointed authorities responsible for the performance of the HBA and project evaluation as a whole, are as such directly impacted by this struggle. In fact, several studies have shown that the AECs in the EU, as well as their non-EU equivalents, appear biased towards promoting the development of research and approving project proposals [66,67,68]. Various factors might contribute to this bias, such as the composition of AECs, often comprised of a majority of the committee members working in animal research [68], a reluctance of members to criticize the work of their peers [67], and difficulties that lay members face when participating in meetings [51,68,69,70]. Additionally, a struggle to change paradigms as described by Kuhn [71,72] may be yet another possible cause. As such, we believe that the ethical consideration of animals within the project evaluation of animal research is likely insufficient if the AECs and HBA are biased against their interests.

#### 3.5.2. Challenge 8: Measuring the Unmeasurable

Another questionable aspect of the method of HBA is that neither harms nor benefits are, in fact, quantifiable. However, this is not an objection that is only made for the HBA in animal research; it is just as applicable to much of the risk–benefit assessments in clinical research, i.e., research with humans. Still, it is probably particularly problematic for animal research [73].

As follows from the principle of proportionality, the weight or significance of conflicting values (see challenges 1 and 2 as described in phase 0 above) must be determined beforehand so that one knows how to balance the harm–benefit scales [74]. This is not possible for, e.g., animals’ subjective feelings of distress or suffering as potential or even likely harms to animals from research, or for future knowledge gains or scientific discoveries further down the line as potential benefits to humans from animal research. Nevertheless, it falls upon the individuals within the AEC to decide how heavy specific harms or potential benefits are or might be.

In HBAs, the severity of harms to animals is assessed at, at least, the following two different time points during animal research: Prospectively by the AEC at the time of the project evaluation or ethical review, that is during phase II, where a classification of severity is assigned, and during the project, that is during phase III (see discussion below) by the researchers, where the actual severity is attributed to each animal. Furthermore, in the following two specific situations, severity must be assessed retrospectively (phase V): for projects with prospective assessment of severe harm and projects involving non-human primates [10]. There is a wide array of tools, training, and methods to identify and assess the severity of animal procedures, several of which are included in annex VIII of the EU Directive. The Directive does provide some guidance for severity classifications. However, different AECs and their members may in practice have very different views of how much suffering, pain, or distress is associated with a certain procedure and how the final degree of severity should (and can) be defined. These differing views imply that, should two different AECs assess the same experiment, it is at least possible that the results of the assessments differ. With this, we do not intend to deny that no two animal experiments are the same, nor do we intend to argue against the necessity of case-by-case severity assessments (as called for in Article 15 of the EU Directive). A harmonization of standards and procedures is nonetheless necessary, in our view, to allow for intersubjective comprehensibility; otherwise, it would be incomprehensible why one AEC comes to result X and another AEC comes to result Y. Additionally, differences regarding results are not necessarily always due to differences in the experiment but could result from differences in the ethical theories, ideas, or views of AEC members.

There are a number of (additional) reasons for the resulting disagreement concerning the weight of harms or benefits, for example, a lack of knowledge in the AEC of animal physiology and behavior or of the procedures and research methods in question, time, and resource constraints, as well as underlying ethical views like speciesist or biased viewpoints amongst committee members, leading to widely divergent concern for different kinds of animals or humans. As such, the outcomes may inevitably differ from AEC to AEC, endangering the internal consistency of the project evaluation process.

#### 3.5.3. Challenge 9: Comparing Apples with Oranges: Animal Harms vs. Human Benefits

Neither are harms and benefits easily measurable (challenge 8), nor are the harms and benefits easily comparable to each other. The HBA requires a balancing of predominantly certain events (harm to animals, to a pre-decided extent) against somewhat predicted but uncertain events (potential benefits derived from research with the ultimate impact of the research being unknown) [75]. Also, as Eggel and Grimm [75] have described, different types of research present distinct potential benefits. Since generated knowledge tends to be seen as holding little legitimating power compared to more tangible promised benefits, the HBA poses a greater obstacle for basic research projects than for applied and translational research [56,75]. Moreover, harms take place at scheduled moments in time while benefits may arise at unknown points in the future or not at all [75,76]. As pointed out by Grimm et al. [56] and further elaborated on by Grimm and Eggel [75], the EU Directive itself explicitly states “may ultimately benefit human beings, animals or the environment” (Art. 38 p.2d [10]). Thus seemingly, and entirely contradictory to the concept of the HBA as a whole, allowing for situations where there is in fact no tangible benefit gained in the end, despite animals having been used. Instead, merely the prospect of achieving a beneficial outcome seems sufficient [75].

Harms are also often of a certain duration and quality (e.g., psychological or physical, severe or mild, acute or chronic, local or general). Benefits, when or if they occur, are not easily categorized in this way. One can thus conclude that, while there is always some uncertainty in assessing harms, there is an exceptionally higher degree of uncertainty in assessing benefits. At the very least, one can state that the HBA performed by AECs therefore seems like comparing apples and oranges [61,76,77,78,79].

To summarize, at present, the ethical review of animal research in the EU and other countries is performed through an HBA, but there are no clear guidelines on how to conduct it. Several authors have proposed methods for performing the HBA, including a combination of discourse and metric models in project evaluation [57]. The discourse model involves a committee of stakeholders engaging in dialogue to deliberate on the expected impact of a particular experiment. Both the anticipated benefits and harms to laboratory animals are considered, with the aim of developing a nuanced understanding of acceptable harm levels for achieving different types of benefits. The metrics model uses predetermined criteria to assign and weigh harms and benefits using a set algorithm. The latter can be conducted without the need for an actual ethics committee [57]. Others have proposed incorporating specific criteria to assess scientific validity, such as the following: construct validity, referring to the level of agreement between the animal model or outcome variable and the quality it is meant to model or measure (essentially, whether the study design can accurately address the scientific question or hypothesis); internal validity, which assesses the adequacy of the experimental design and the extent to which the study minimizes bias and confounding variables; and external validity, which pertains to the inference space of a study, that is, the range of conditions and populations to which the findings can reliably be generalized [80,81]. However, as described by Eggel and Grimm [75], there is no unanimously recognized method for performing HBA. Even across the EU, no uniform approach to conduct HBA is used, since there are no clear guidelines to be found in Directive 2010/63/EU [10]. Thus, the HBA remains a challenge. The search for a new framework for the ethical assessment of projects involving animals seems inevitable and should be prioritized in animal research ethics.

### 3.6. Challenges of Phase III: Conduct of Animal Research

Following the detailed assessment of ethical, legal, and practical challenges associated with the ethical, legal, and social presumptions (phase 0), planning (phase I), and review (phase II) of animal research, we are henceforth describing the challenges associated with the remaining four of the seven phases of animal research. The following discussions of these phases partly build upon those already considered.

For the discussion of phase III, we are highlighting the following two challenges: “Protecting the wellbeing of animals during research” and “Ensuring the prospect of scientific benefit, e.g., by preregistration”.

#### 3.6.1. Challenge 10: Protecting the Wellbeing of Animals during Research

In conducting animal experiments, there is at least the challenge of considering how actual harm should be classified and assessed, which points to the strong connection between phase II as described above and phase III. Though there are notable efforts to decrease the general lack of knowledge about how laboratory animals experience certain interventions and conditions and what is (therefore) harmful to them (and to what extent) [82,83], the actual assessment of welfare remains challenging. This leaves open the possibility of disregarding their suffering and distress, especially mental and emotional distress, due to the lack of knowledge about their mental lives [84].

In this context, the problem of the power imbalance between the animals used for research and the humans involved in this research also becomes apparent. This imbalance manifests in various ways, e.g., humans interpreting animals’ expressions and determining not only their meaning in a given situation but—crucially—also establishing if an animal has (or does not have) the capacity to understand the situation and its implications and is thus (not) able to make a decision for itself or at least contribute to decision-making by assent or dissent (see challenge 3 of phase 0 above). Another manifestation of power imbalances in the context of research is the problem of so-called adaptive preferences ([85], p. 343). Animals raised specifically for the purpose of research usually have no experience of a life outside of this specific environment and the development of their preferences is therefore severely restrained. Consideration of animal assent and dissent might nevertheless be helpful for improving the conditions of research animals within the existing paradigm of animal use [38]. As such, they can play a role in designing research involving animals to further reduce its harm.

For animal research to be (more) respectful of animals as research subjects, legal requirements for research with animals need to be reformed to further protect their well-being and interests. This need for increased legal protection aligns with a need for more knowledge about animal interests, particularly those arising from sentience if the respective animal or species can be classified as sentient. A promising start for both seems to be to model them on the requirements for ethical research with humans, as suggested by some scholars [38,86]. The resulting legal challenges can be tackled by calling into question the current legal status of animals [34].

#### 3.6.2. Challenge 11: Ensuring the Prospect of Scientific Benefit, e.g., by Preregistration

Another specific challenge of phase III relates to the scientific methodology used in handling and overseeing research with animals, since only if the appropriate methods are chosen and correctly applied, then there is even a prospect of scientific benefit (potentially benefit to humans) that could result from research with animals. Apart from reinforcing and improving the validity or robustness of animal research in general (e.g., [80,81]), the preregistration of animal research studies and of the planned methodology has also been proposed as one measure to address the scientific challenges associated with conducting qualitatively “good” animal research (see, e.g., [87,88]). However, the call for the preregistration of a study comes with its own challenges, especially relating to the question of how to encourage researchers to preregister their studies to ultimately contribute to improving the quality of animal research and, with this, the likelihood of scientific benefits to humans. This relates to phase IV (Publication/Dissemination of Results of Animal Research) as preregistration might be used as a requirement for publication by publishers to incentivize this, in our view, ethically required measure to address the challenge of conducting research that can benefit science.

### 3.7. Challenges of Phase IV: Publication/Dissemination of Results of Animal Research

Since typically only published findings can find their way into the body of evidence (which contributes to justifying that animals are used for research at all), this phase is crucial. The following two challenges require special attention: “Improving reporting quality” and “Publication bias to only publish positive results”.

#### 3.7.1. Challenge 12: Improving Reporting Quality

To improve the quality of reporting, the ARRIVE guidelines (Animal Research: Reporting of In Vivo Experiments) were introduced in 2012 and updated in 2020 [89,90]. In the latest version, 21 items are listed for orientation during manuscript preparation, starting with “Study design” and ending with “Declaration of interest” [89]. More than 300 academic journals have endorsed these guidelines. However, even after the introduction of these guidelines, authors, reviewers, and journal editors did not always properly implement them [91].

**Figure 3:**
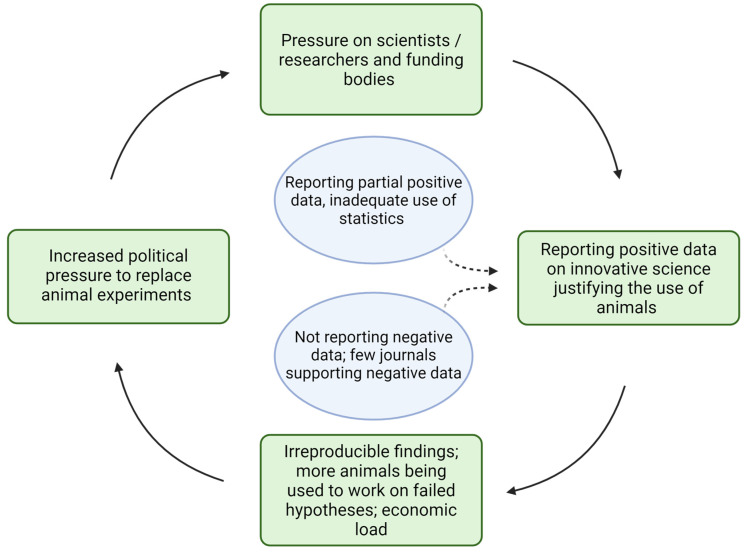
A vicious cycle that leads to more rather than less animal testing.

Only a minority of scientists are trained in reporting accurate statistics. Also, some journals do not emphasize reporting a method for statistical power (i.e., the probability of a statistical test for detecting an actual effect in a population; it depends, among other things, on the sample size but also on the statistical method used) and sample size calculations that ensures there is a sufficient sample size in the experimental design. This is important to detect an effect of treatment and to prevent type I errors (false positives). Descriptive statistics and parametric tests (which often assume a normal distribution of the data) are used instead of non-parametric tests (that make no assumptions about the distribution) on a dataset with a small sample size, which further complicates the situation [92,93,94,95]. Moreover, the methods involved in randomization and blinding are not described in detail in 70–80% of high-quality journal articles and, to focus on technically difficult and innovative science, some journals disregard the fundamental standards of experimental design and data analysis. As a result, they promote irreproducible findings and establish low standards for clinical translation, giving rise to the conflict as to whether animal experiments should be completely replaced due to their inability to be replicated and their inability to be translated into humans [91,93] or whether more animal research needs to be conducted to prove its usefulness and its ability to create positive results (see Figure 3 below; see also the next challenge).

#### 3.7.2. Challenge 13: Publication Bias to Only Publish Positive Results

The research community has grown accustomed to praising only positive findings, i.e., findings that support the tested hypothesis. Few journals, e.g., Journal of Negative Results in Biomedicine and Journal of Pharmaceutical Negative Results, explicitly support the publication of negative results [96]. Negative results—i.e., findings that do not support the tested hypothesis—could, in case of attempts to replicate the previous studies, be attributed to the use of different reagents or conditions, a lack of statistical power, or the complexity of the biological system, making it difficult for scientists to make reliable statements about the hypotheses. Publishing negative results is unattractive to scientists, as it can impede a fellowship or funding for continued research, as they may not be of interest to the lab head, and as it is still arduous and time-consuming to undergo a complicated review process for publishing in journals reporting negative findings [97].

Failure to publish negative results, however, contributes to misguided projects receiving financial support and being retested, producing (further) negative results, and potentially also resulting in the use of more animals for research than would be used if negative results were published.

### 3.8. Challenges of Phase V: Further Exploitation of Published Results of Animal Research

The publication of the results and all relevant background information on the study can be considered a milestone in the realization of the potential benefits that legitimize the animal experiment. The results of individual projects often form pieces of a larger picture with, however, remaining uncertainty and a lack of control over the ways the animal research results will be used in the future. We want to highlight only one, overarching, challenge here, which we call “the vicious cycle of animal research”.

However, in addition to this challenge, further challenges might emerge, such as animal research after publication (or potentially even prior to being published) being misused for unintended purposes. An example of this is animal research that is classified as dual-use research of concern [98]. As this potential for misuse is not specific for animal research, we have not included it as a challenge here. Another problem that can be associated with animal research is that the animal research might result in products harming animals, humans, or entire environments in an ecotoxic way. This is also not specific for animal research, as non-animal research might result in ecotoxic products as well.

#### Challenge 14: The Vicious Cycle of Animal Research

The focus placed on phase V for the evaluation of potential benefits can become a problem if undesirable side effects are not considered and measures are not taken to contain them. Increased political pressure from citizens and government bodies to completely replace animal experimentation [99,100,101,102,103,104] also puts pressure on scientists conducting animal research to report innovative science and interesting positive findings, thereby justifying the use of animals in research. To receive funding support for ongoing research, scientists are not focused on reporting negative findings, and sometimes partially positive results are reported with inadequate use of statistics and poor peer review process, which frequently results in failed clinical translation and promotes irreproducible biological findings (see phase IV). This reproducibility crisis contributes to the political pressure to justify animal research. Consequently, and based on some authors’ own experiences and concerns as researchers, we tentatively stipulate the existence of a vicious cycle that encourages more animal experimentation rather than reduction (see Figure 3). A comparable vicious cycle can exist regarding non-animal methods (we thank an anonymous reviewer for pointing this out); but this does not change the problem of such a cycle when it comes to animal experimentation.

### 3.9. Challenges of Phase VI: Evaluation of Animal Research

The final phase of animal research relates to its overall evaluation. In an overarching sense, this includes evaluating ethically but also scientifically aspects of the results or the method. The following main challenges are described in the subsequent two subsections: “The need for evaluation of the accountability of the HBA”, and “Evaluating animal research as a whole—animal free alternatives” (see also challenge 6 in phase I: “Refraining from planning animal research—the search for alternatives”). It is important to note that phase VI (overall evaluation of animal research) and phase V (further exploitation of animal research) are likely taking place in parallel. We decided to put phase VI last because of its overarching character and because phase V might not apply to every single animal study, whereas phase VI (ideally) does.

#### 3.9.1. Challenge 15: The Need for Evaluation of the Accountability of the HBA

As previously described, regarding individual projects, retrospective assessment is mainly performed when projects have been classified as severe at the outset [10]. As illustrated in a study by Pound and Nicol [105], a diminishingly small amount of animal studies approved based on an HBA were, when put through a corresponding weighing of harms and benefits after completion of the animal studies, assessed as permissible according to the demand that the benefits balance out the harms. This raises serious questions about the reliability of the HBA and ethical review process and stresses the importance of increased retrospective assessments to both increase transparency and accountability of using the HBA as a tool for ethical decision-making in this context.

#### 3.9.2. Challenge 16: Evaluating Animal Research as a Whole—Animal-Free Alternatives

Changing the established system of the use of animals in experiments is time- and material-intensive. Availability and limited selection in animal product replacements (see discussion above related to challenge 6 in phase I) are creating a monopoly for those products and high prices. Low demands for such products in general further reduce the willingness to invest in the development and production of replacements as well as selling them for a reasonable price to global players. As a result, animal experiments can appear to be without alternative.

Yet, progress regarding novel testing systems (e.g., microphysiological and microfluidic devices) opens opportunities to conduct real animal-free research [106,107]. In general, to state that such a model system withholds the power to replace animal models is misleading. However, what can currently be achieved with microphysiological systems is not yet comparable with in vivo conditions, i.e., tests on living animals. The replacement technology is still in its infancy, aiming to rebuild the core functions of organs and/or tissues. Instead of replacing animal experimentation, these systems could currently represent a powerful tool aside from in vivo investigations. The potential of these systems as drug screening platforms, especially for toxicity testing, has already been shown and is currently being considered by the FDA as a new approach methodology/non-animal method (NAM) for predictive preclinical studies [108,109]. For basic research, the application of such a model allows a focused view within a highly controlled environment [110]. Taken together, opportunities to evade the implementation of animals for research and the application of deriving animal-free materials are both increasing. However, challenges remain. If a scientist prefers going animal-free in research, it is a question of which research field one works in and especially of one’s dedication, the degree of commitment that one feels towards such a decision, and eventually also a matter of financial support.

### 3.10. Further Challenges

The following challenges describe topics that were addressed during the retreat week, but which we want to keep shorter, more summarized, and more hypothetical here; we refrain from a strict allocation to the phases, also because they sometimes address several phases or concern overarching aspects of ethics in connection with our treatment of animals.

Animal experiments and their practical ethical evaluation are often an example of “ethics within legal constraints”. This applies in particular to the project evaluation (ethical review) by an AEC. This is because, in contrast to a purely philosophical approach that can also ignore existing legal requirements, the ethical review can only take place against the background of an existing legal framework. As legislation allows for the using and harming animals for research purposes, ethical aspects to be included in the review are thus limited to ones that do not challenge this status quo, which typically relies on a utilitarian—or consequentialist—and anthropocentric rationale (see also challenge 2 in phase 0 above). Some authors view this orientation towards utilitarian thinking as problematic [3].

It is true that utilitarianism does not recognize any a priori defined limits to action (as is, for example, the case with protection through the attribution of inherent value) but makes this dependent a posteriori on the morally relevant consequences (e.g., how much pain or happiness or preference fulfilment an action generates for all the moral entities that are affected by this action). This can hypothetically make it possible for the interests of a minority (here: animals) to be “sacrificed” in favor of the interests of a majority (here: humans) but not necessarily. Current legal practice for AECs is therefore by no means congruent with (all) the utilitarian theories or their applications.

Still, it is argued that any ethical theory or deliberation which would lead to other conclusions is as such deemed inconsistent with the applicable law or inappropriate by the legal framework that itself demands but does not elaborate on, “taking into account ethical considerations” (Article 38 p. 2d [10]). Thus, ethical discussions by AECs are as such truncated to suit the notion that animal use is, in principle, ethically acceptable since it is legally acceptable. Therefore, in the given legal framework and established AEC practice, one could criticize, overarching ethical questions such as the following are mostly disregarded: Do animals have a right not to be subjected to pain caused by humans at all? Is it ethically acceptable to breed, keep, use and kill animals for research? Instead, the ethical weighing is forced to operate within a “Given that animal research is legitimate” mindset and the questions allowed are limited to ones such as the following: How much pain can we justify? How many animals are we allowed to kill [111]? From a philosophical perspective, the ethics within the ethical review is as such limited and probably inherently skewed from the start. However, it is true that an AEC or a specific ethical review of an experiment cannot be expected to address such fundamental questions every time; there are other institutional bodies and places for social discourse for this purpose. But as long as morality is still to be taken seriously, a rational person cannot completely ignore these questions. The issue is therefore how such a limitation of ethical reflection can still be dealt with responsibly in the context of an AEC or ethical review, and how possible negative effects on decision-making could be countered by excluding the issues due to the legal framework.

Other overarching questions relating to the ethics of animal experimentation concern the fundamental considerations about the relationship between humans and animals. These also characterize our thinking about animal experiments. Some scholars argue that, historically, the perspective on animals has been intertwined with aspects of domination, considering how society has implemented a form of violence against animals that fundamentally shaped the human–animal relationship [112]. One should also take into account a broader perspective on the relationship between humans and animals and the related (philosophical) positions, such as a general vegetarian or vegan lifestyle. If one considers such a position, which attempts to reduce or even avoid suffering in animals, one must nevertheless critically raise the question of whether there really is the possibility of eating without causing harm, albeit indirectly, to the environment, to animal or human life; the realization of a life with “zero impact” seems to be a regulatory ideal that we can think of reaching as an asymptote [113,114]. Accordingly, one could also argue that the idea of completely animal-free research can only be a regulative ideal that we can approach but which we cannot achieve, and we must accept that we humans in the anthropocene will never be able to lead a life that avoids any harm to animals. Nevertheless, this of course does not prevent us from trying to improve our relationship with animals as much as possible in order to approach the asymptote as closely as possible. An important challenge of animal (research) ethics from a philosophical point of view is then to clarify the more fundamental relationship that we humans have with animals and also to scrutinize possible problematic views of this relationship, as these can have an imprint on the ethics of animal experiments; our attitudes towards intensive farming can also influence our attitudes towards animal experimentation, or rather both can be based on a certain understanding of the relationship between humans and animals.

Such attitudes can also play a role with regard to further and more mediate consequences of animal experiments for the animal and non-animal environment; as briefly mentioned in Section 3.8 (Challenges of Phase V) above, animal research could result in pharmaceuticals that might be ecotoxic to the environment. It is important to highlight this challenge since, in this article, we are focusing on the use of animals in biomedical research (see Introduction). The explicit aim of this type of animal research precisely is the development of therapies, often in the form of pharmaceuticals. It should therefore not go unnoticed that a “successful” (series of) animal experiment(s), the results of which are the marketing of new pharmaceuticals, might ultimately result in the introduction of many of these pharmaceuticals—those which are not used but discarded—into the environment. Since such discard might have a toxic effect on ecosystems, this results in the further need for animal research to specifically test the ecotoxicity (i.e., toxicity to the ecosystem) of pharmaceuticals in order to avoid harm to ecosystems and by this nonhuman and human animals [115].

### 3.11. Challenges and Opportunities Related to the Conception of Animal Research Ethics as a Field

As shown, animal research ethics faces various challenges across multiple domains. We have thus far examined some pertaining to the subject area itself, i.e., animal experiments, where various ethical, legal, scientific, and practical issues can be located throughout all phases of an animal experimentation project. However, yet another set of challenges has arisen with the unfinished development of this emerging field: Establishing a suitable set of methods for animal research ethics, working out disciplinary boundaries, building an interdisciplinary body of knowledge, and fostering collaboration among groups with conflicting viewpoints. Moreover, there are also debatable questions concerning the goals and central assumptions of animal research ethics. It is also still unclear how animal research ethics can sensibly but also practically deal with (meta-)theoretical challenges, such as ethical pluralism. This section characterizes a few of these challenges associated with animal research ethics as a field but also shows ways to address the issues and thereby points to the opportunities of this emerging field. It contains the following four subsections: “Moral pluralism in animal research ethics”, “Beyond the laboratory: animal research ethics in unexpected places”, “Internationality: Plurality in codes of conduct, standards, and legal norms”, and “Learning from each other: interdisciplinary education in animal research ethics”.

#### 3.11.1. Moral Pluralism in Animal Research Ethics

Animal research ethics should take seriously the aforementioned problem of pluralism in theory (see challenge 1 of phase 0). There are different streams of animal ethics itself, reaching from welfare increasing measures to animal rights approaches. Consequently, a key challenge in the development of an international and interdisciplinary animal research ethics is finding and establishing a shared normative position or to find a way to accept moral pluralism. This involves addressing questions regarding the consideration of interests or status of animals, their moral significance, and how they relate to other goods, such as scientific freedom or human health. The inherent plurality of sometimes incommensurable norms necessitates that animal research ethics forms a position on the issue; pluralism might either be rejected or embraced. It can be rejected either by dissolving pluralism within a monistic account of morality—that is, by developing the “correct” normative ethical theory—or by choosing which way of looking at the animal is the right one, i.e., what norms we should prioritize. Both strategies face obvious difficulties since developing a monistic account requires solving a fundamental problem in ethical theory that seems far from being solved, and a choice faces the critique that it is not clear who should have the burden, responsibility, and the mandate to choose the right account.

If, on the other hand, animal research ethics decides to embrace pluralism, it will have to face the following fundamental issue: How are we supposed to decide in each situation? A descriptive account of ethics that embraces the existing pluralism in animal ethics might describe the variety of norms as an aid in dealing with morally problematic situations [116] but would not provide clear guidance or solutions for any moral conflict. Again, there would remain the problem of who should bear the responsibility to choose, which might leave the burden of moral judgement to AEC members, where it currently lies. While AECs are bound by the law, their task is morally far more demanding than simply “applying” existing law: “The standard of ethical decision-making for the majority of commission members was their intuition and personal conviction: They most frequently proceeded according to their intuition/their personal moral feeling […].” ([117], p. 71; own translation).

#### 3.11.2. Beyond the Laboratory and Beyond Mere Reflection: Tasks of Animal Research Ethics

Of course, animal ethics and research ethics have long been researching ethical issues relating to animal experiments, formulating recommendations and actively advocating changes. Nevertheless, the understanding of the role of ethics in relation to animal experiments depends on each individual person. If we want to understand animal research ethics as a field that is interdisciplinary in structure, it is also necessary to be clear about the tasks that are to be dealt with in such a field.

So, when it comes to the range of tasks of an animal research ethics, the following two things seem important to us: firstly, that the task must not only be to look at the laboratory where the animal experiments take place, so to speak and, secondly, that not only ethical questions of justification may be dealt with. Animal research ethics should thus have to take into consideration the scientific, legal, regulatory, societal as well as (sometimes) political views on research with animals. As scholars of an applied discipline, animal research ethicists should ideally not just reflect on but also inform the practice of animal research in the laboratory as well as in regulatory institutions and societal discourses. This may imply a broader concept of ethics that goes beyond the mere discussion of ethical justification, i.e., contributing to a normative discourse. It can include interdisciplinary and empirical research activities but also communication and outreach activities that go in the direction of what is currently being discussed in the context of “translational bioethics” [118,119].

Based on the discussions above, we suppose a clear ethical judgement can be made which suggests the need for changes or even a paradigm shift in the current practice of animal research. To achieve such a change or shift, for example, one goal of a (future) international animal research ethics could be to reduce the number of animal experiments overall. This can be justified by both animal ethics considerations and research ethics considerations such as the problem of the vicious cycle discussed above (challenge 14 in phase V), which encourages scientists to conduct more animal experiments rather than reduce their number. Implementing such a change requires more than just compelling ethical arguments. It requires changes in legislation, enough political traction, and support from the public. However, people have very different intuitions about animal ethics [120], which shapes political developments.

If we assume that not just the researcher conducting experiments on animals and the policymaker responsible for enacting regulations have a responsibility towards animals but also the scholar of animal research ethics, then it is necessary for a scholar of animal research ethics and thus for animal research ethics as a field to pay due regard to the systemic and societal realities of animal research and to the conditions for transformation. This means, for example, focusing on policymaking processes which determine the limits of animal use, on the role of the public discourse, and on the psychological underpinnings of anthropocentrism (see challenge 2 in phase 0). This perspective opens questions on the role of practices such as science communication, engagement with policymakers, and activism within animal (research) ethics. Since all of this has an impact on animal research, it could—or perhaps even should—also lie within the realm of animal research ethics to reflect on and inform these activities.

#### 3.11.3. Internationality: Plurality in Codes of Conduct, Standards, and Legal Norms

Incorporating ethical principles and insights into guidelines for practice presents its fair share of complexities.

As shown in various phases (see especially challenges 8–11 in phase II and phase III and “Further challenges”), there are numerous limits to the currently used HBA. So, where do we go from here? A project evaluation or ethical review of animal research will always be constrained by the legal framework within which it operates (see “Further challenges”) and could therefore either be revised or replaced within this framework, or the legal framework could be revised or replaced. Time and funding could also be directed at creating another more comprehensive, user-friendly, and widely applicable tool. Acknowledging the limits of an HBA, many suggestions for other ethical decision-making models have been made [57,80,121,122,123,124,125,126]. The task of establishing a shared or even just a single cohesive position for an international and interdisciplinary animal research ethics is not a simple feat, as illustrated by the following examples.

Reconciling several different tools of animal research ethics is challenging. Petkov and colleagues [30] made a proposal for an updated Basel Declaration that combines different ethical principles to lay a foundation for international collaborations (see discussion of challenge 3 “Operationalization of ethical principles” in phase 0). The authors assume that principles such as the 3Rs [32] and the 6Ps (three principles for social benefit and three principles for animal welfare, including an HBA [5], are complementary [30]. But it is also not far-fetched to claim that ethical principles or positions such as the abolition of (almost) all animal experiments, the obligation of their reduction, and justifying them via the social benefits (for humans) contradict each other because they come from different ethical traditions or schools.

Existing international guidelines are not satisfactory. As shown elsewhere [27,33], the existing Basel Declaration gives human interests a priority over animal interests, and animals are protected only in as much as the 3R principles are endorsed there. Due to the fact that Russel and Burch [32] have neglected to include ethical theory in their writing, it is open for debate if or to what degree the 3Rs are based on the ethical motivation of protecting animals or are rather motivated primarily by a scientific rationale of improving science and research results [33]. Perhaps this debate over the original intention of the 3Rs could have been avoided if Russel and Burch had included a consultation of ethical expertise in an interdisciplinary research group.

Navigating legal pluralism can also be challenging. As illustrated above (see challenges 1 and ”Further Challenges”), the many differences between legislation and practices across countries and continents pose a serious limitation to (more) international animal research ethics. To give another example, there currently exist (according to our own experiences, at least) significant differences in the duration of the review of research proposals in different countries. If authorities that approve animal experiments work according to internationally uniform criteria, these differences in approval time that can be a problem for international competition in research can be prevented.

Implementing greater international uniformity in animal research can, however, also be a cause for new problems. This can be illustrated by Directive 2010/63/EU [10]. According to Article 2 (1) of the Directive, member states are permitted to retain stricter national measures than those provided by the Directive. If a member state had stricter animal welfare standards in place before the Directive came into force, it could maintain these standards. However, pursuant to Article 2 (2), member states are obligated to allow the import and use of animals from the other EU member states, if they have been bred and maintained in compliance with the Directive. This also encompasses products derived from animal research that aligns with the Directive even if stricter national regulations on animal welfare are in place (and were established before the Directive was issued) in the receiving country. The endeavor to preserve international competitiveness in research and in the development of (medicinal) products thus prohibits member states from enacting stricter regulations regarding the import. It can be anticipated that, as regulations on animal research become more standardized internationally, more countries may be inclined, though not obliged, to relinquish existing stricter regulations to ensure international competitiveness and promote trade.

The more uniform the standards, the less possible it becomes for local governmental bodies to oversee animal research and maintain “higher” standards since enforcing higher moral standards by national law would breach international standards a country has agreed to. As said, for example, if an EU member state prohibits the import of products developed in other countries under more liberal laws than those in the member state, it could be in violation of the Directive. This violation would occur regardless of whether the member state’s motivation is economic or moral.

On the other hand, the internationalization of animal research ethics could help to identify and (ideally) avoid mistakes from one country in another. As an example, Svea Jörgensen and colleagues conducted a study identifying several problems with how HBAs are conducted (addressing challenge 15 “The need for evaluation of the accountability of the HBA” in phase VI) in Sweden [49]. The publication of these results in a journal with an international readership (in this case, *Animals*) may help to avoid similar problems abroad.

In addition, in theory, creating uniform standards on how ethics should be involved within the different phases of animal research and on how research with animals should be regulated legally could help with avoiding “outsourcing” research that would not conform with standards in one jurisdiction to places on earth with more lax standards. This is only possible, however, in theory and not the best way to address “ethics dumping” at the time being. This is because “ethics dumping” could certainly be eradicated if standards were uniform across all countries worldwide. Such a harmonization is, however, indeed utopian. It would require a unified legal framework in the form of an international agreement based on a minimum consensus among state parties and would certainly not be an application of EU law worldwide. Because of these limitations of harmonizing regulations, alternative ways to address “ethics dumping” should be discussed.

Despite the limitations, it should still be encouraged to internationalize animal research law as much as possible within the EU.

#### 3.11.4. Learning from Each Other: Interdisciplinary Education in Animal Research Ethics

Because the challenges described so far concern many disciplines, including especially ethics, law, politics, economics, biology, and medicine, an interdisciplinary perspective in animal research ethics is crucial. It is evident that interdisciplinary approaches to animal research ethics are necessary due to the fact that various domains (such as laboratory research, medicine, ethology, politics, law, ethics, and sociology) intersect within the challenges that concern this emerging field.

One major obstacle when it comes to intertwining a study with these different aspects under animal research ethics, however, is strongly connected to the differing levels of knowledge of the scholars and other actors involved. If interdisciplinarity could be embedded early on during academic training, it could potentially strengthen collaborative efforts and knowledge transfer within the field. By the same token, an interdisciplinary animal research ethics has the potential to bring natural sciences and humanities closer to each other.

Although empirical and descriptive pursuits are regarded as the core of natural sciences, the final goal and motivation of science as such, also in the case of (biomedical) animal research, is a normative one; animal research deals with morally highly relevant goods, viz. human and animal health. This connects biomedical research or generally natural sciences with ethics, philosophy, and other humanities. Unfortunately, due to the historical development of academia, especially the differentiation into increasingly less interconnected branches such as sciences and humanities (the “two cultures”, as Snow [127] classically termed them), these disciplines have separated to such an extent that the training in animal research hardly involves ethical thinking that goes beyond established animal welfare standards. In turn, (animal) ethics sometimes leans towards rather hypothetical or speculative thinking and may fail to systematically gather and methodologically understand not only the empirical results about animal welfare and animal minds (see challenge 8 “Measuring the unmeasurable” in phase II) but also the practical problems arising in the different phases of animal research. Cherry-picking of evidence should be avoided.

Thus, scholars of animal (research) ethics as well as animal researchers could benefit from an education that contains at least fundamentals of both domains. A well-rounded training in ethics provides methods, concepts, and terminology to reflect about normative assumptions, which are often implicitly present or only stated—and not substantiated—in scientific papers. Consequently, natural sciences rely, to some extent, on ethics and philosophy to contemplate normative questions. The lack of a shared foundation of education in ethics and science could be a hurdle for the field of animal research ethics. Conversely, ethics needs practical description, including empirical data, on what is carried out in a particular animal experiment and what is required to reach sound conclusions. Therefore, it may be valuable for scholars from animal ethics to also be educated in relevant topics of natural sciences.

That being said, the methods of rational thinking and logical argumentation are part of every science, and therefore animal research scientists and ethicists already share, irrespective of all differences, at least a common methodological ground.

## 4. Conclusions

We started with the following question: (How) can non-human animals be used for the benefit of humans in a manner that is both scientifically and morally justified? In our view, this question can only be answered from the perspective of animal research ethics. We understand animal research as a complex process and suggested a model of seven phases to categorize the ethical, scientific, and legal challenges. In this context, we understand animal research ethics as an interdisciplinary and international endeavor.

The results of an international retreat week on animal research ethics, which have been compiled and categorized here, can only represent the beginning of a further examination of the various questions and challenges animal research ethics is phased with. In addition to the goal of primarily presenting the challenges and selected opportunities of animal research ethics but, for the most part, not (yet) providing answers, this article has also some limitations. Given its scope, a systematic literature search was not undertaken. Therefore, it should not be regarded as an exhaustive resource on all issues pertaining animal research ethics that have already been discussed. It was also not possible to cover all challenges in equal detail. This was due to the composition of the participants, the prioritization of challenges and the time available. Nevertheless, we think it is important to provide an overview. Still, further research is needed, including a systematic and/or more comprehensive narrative review to create a comprehensive account of challenges surrounding the ethics of animal experimentation and, of course, a more detailed analysis of each challenge.

The phase model (see Figure 1) utilized in this article provides an application-oriented structure into which existing and future research—be it theoretical or empirical— could be integrated. The advantage of this model lies in its capability to detail the practical realities of animal research, while also allowing for a subsequent examination of the interdependent or systemic aspects of the ethical challenges that are described in detail in Section 4 and have been summarized in Table 1.

In this regard, the article lists discussion points for the further development of animal research ethics and does not represent a “finished” concept with broad expert consensus; this task still lies ahead for the field.

## Figures and Tables

**Table 1:** Summary of the challenges of animal research viewed from animal research ethics.

PHASES OF ANIMAL RESEARCH AND KEY CHALLENGES	
**Phase 0: Ethical, legal, and social presumptions of animal research** (see Section 3.3)	
Normative pluralism Anthropocentrism and the perspective of animals	3. Operationalization of ethical principles 4. Animal rights and consent
**Phase I: Planning of animal research** (see Section 3.4)	
5. Comparability to human conditions when using animal models in medicine	6. Refraining from planning animal research–the search for alternatives
**Phase II: Research review of animal research proposal(s)** (see Section 3.5)	
7. Insufficient considerations for animal interests in the tools of ethical deliberation	8. Measuring the unmeasurable 9. Comparing apples with oranges: animal harms vs. human benefits
**Phase III: Conduct of animal research** (see Section 3.6)	
10. Protecting the wellbeing of animals during research	11. Ensuring the prospect of scientific benefit, e.g., by preregistration
**Phase IV: Publication/Dissemination of results of animal research** (see Section 3.7)	
12. Improving the reporting quality	13. Publication bias to only publish positive results
**Phase V: Further exploitation of published results of animal research** (see Section 3.8)	
14. The vicious cycle of animal research	
**Phase VI: Evaluation of animal research** (see Section 3.9)	
15. The need for evaluation of the accountability of HBA	16. Evaluating animal research as a whole–animal-free alternatives

## Data Availability

Data are contained within the article.
