# Correction: Mertz et al. Interdisciplinary Animal Research Ethics—Challenges, Opportunities, and Perspectives. *Animals* 2024, *14*, 2896

**DOI:** 10.3390/ani15121803

**Published:** 2025-06-19

**Authors:** Marcel Mertz, Tatiana Hetzel, Karla Alex, Katharina Braun, Samuel Camenzind, Rita Dodaro, Svea Jörgensen, Erich Linder, Sara Capas-Peneda, Eva Ingeborg Reihs, Vini Tiwari, Zorana Todorović, Hannes Kahrass, Felicitas Selter

**Affiliations:** 1 Institute for Ethics, History and Philosophy of Medicine, Hannover Medical School, 30625 Hannover, Germany; 2 Section Translational Medical Ethics, National Center for Tumor Diseases (NCT) Heidelberg, Department of Medical Oncology, Heidelberg University Hospital, Medical Faculty Heidelberg, Heidelberg University, 69120 Heidelberg, Germany; karla.alex@med.uni-heidelberg.de; 3 Department of Law, Freie Universität Berlin, 14195 Berlin, Germany; 4 Department of Philosophy, University of Vienna, 1010 Vienna, Austria; samuel.camenzind@univie.ac.at; 5 Department of Philosophy and Humanities, Freie Universität Berlin, 14195 Berlin, Germany; 6 Humanities Department, Università della Calabria, 87036 Rende, CS, Italy; 7 Department of Applied Animal Science and Welfare, Swedish University of Agricultural Sciences, 750 07 Uppsala, Sweden; 8 Messerli Research Institute, University of Veterinary Medicine, 1210 Vienna, Austria; 9 Vienna Doctoral School of Philosophy, Department of Philosophy, University of Vienna, 1010 Vienna, Austria; 10 i3S–Instituto de Investigação e Inovação em Saúde, Universidade do Porto, 4200-135 Porto, Portugal; sara.capas@i3s.up.pt; 11 ICBAS School of Medicine and Biomedical Sciences, University of Porto, 4050-313 Porto, Portugal; 12 Karl Chiari Lab for Orthopaedic Biology, Department of Orthopedics and Trauma Surgery, Medical University of Vienna, 1090 Vienna, Austria; eva.reihs@meduniwien.ac.at; 13 Institute of Neuronal Cell Biology, Technical University Munich, 81377 Munich, Germany; 14 German Center for Neurodegenerative Diseases, 81377 Munich, Germany; 15 Graduate School of Systemic Neurosciences, Ludwig-Maximilians-Universität, 82152 Planegg, Germany; 16 Department of Philosophy, University of Belgrade, 11000 Belgrade, Serbia; 17 Center for the Study of Bioethics, 11000 Belgrade, Serbia

## Corrections in Simple Summary and Abstract

In the original version [1], the simple summary and abstract incorrectly referred to 20 key ethical, legal, and practical challenges. **These 20 were condensed to 16 during the last revision of the manuscript**; in the main text, i.e., Table 1 (Summary of the challenges of animal research viewed from animal research ethics) the number was correct. Accordingly, the text passages now read as follows:

“Here, 16 key ethical, legal, and practical challenges are identified and analyzed that need to be addressed.” (Simple Summary)/“In total, 16 key ethical, legal, and practical challenges that an ethical framework for the use of animals in research needs to address are identified and analyzed.” (Abstract).

## Minor Typesetting Corrections

In the course of the major corrections regarding missing citations (see below), **we also have corrected two minor typesetting errors**: 

3. Results, 3.1. Some Regulatory Challenges Associated with Animal Research: “The directive proclaims the need for animal protection […]” was changed to “The Directive proclaims the need for animal protection […]” (the D in “directive” was capitalized).

3. Results, 3.4.2. Challenge 6: Refraining from Planning Animal Research—The Search for Alternatives: “When striving to safeguard the methods and results, it is important to keep in mind, however, that for each laboratory animal calculated, many times more animals must be produced” was changed to “When striving to safeguard the methods and results, it is important to keep in mind, however, that for each laboratory animal calculated, many times more animals must be produced.” (period at the end of the sentence was missing).

## Missing Citations

When converting the original references into numbers for the manuscript, there have been shifts, so that in the original [1] (uncorrected) version the reference numbers are incorrect, mainly from citation No. 21 onwards. These shifts have also resulted from the fact that the following references, which were present in the original text, were overlooked in the conversion to the manuscript and had to be added again.

In the original publication, **WMA (2024) (Ref. 29)** was not cited. The citation has now been inserted in **3. Results**, **3.3. Challenges of Phase 0: Ethical, Legal, and Social Presumptions of Animal Research**, **3.3.3. Challenge 3: Operationalization of Ethical Principles in Legal Norms** and should read as follows:

“Following the Declaration of Helsinki from the World Medical Association for human subject research [29], according to Petkov et al. [30], an amended version of the 2010 Basel Declaration (“A call for more trust, transparency and communication on animal research”) for animal research [31] should serve as a more complete animal research declaration, which includes a unified set of principles.”

In the original publication, **Home Office (UK) (2023) (Ref. 46)** and **National Research Council (US) Committee for the Update of the Guide for the Care and Use of Laboratory Animals (2011) (Ref. 48)** were not cited. The citations have now been inserted in **3. Results**, **3.5. Challenges of Phase II: Ethical Review of Animal Research Proposals** and should read as follows:

“This phase encompasses challenges that both the scientists submitting the animal research proposal and the members of the Animal Ethics Committee (hereinafter referred to as “AEC”) assessing the research proposal face (such committees can also have different names; UK: Animal Welfare and Ethical Review Body (AWERB) [46]; EU: Animal Ethics Committees (AECs) [10]; USA: Institutional Animal Care and Use Committees (IACUC) [47,48].”

In the original publication, **Grimm et al. (2017) (Ref. 56)** was not cited. The citation has now been inserted in **3. Results**, **3.5. Challenges of Phase II: Ethical Review of Animal Research Proposals** and should read as follows:

“Although all the above concerns are important, we argue that the most pressing issue to deal with is that the tool provided—and, e.g., in the EU, is legally required to be used—for the ethical review, the HBA is, in fact, alarmingly often unfit for its intended purpose [56,61].”

In this context, we later also adjusted the citations of Grimm et al. in **3. Results**, **3.5.3. Challenge 9: Comparing Apples with Oranges: Animal Harms vs. Human Benefits** and slightly revised the text passage so that it corresponds better to the now inserted reference. We have also removed the remark “emphasis added” regarding the direct quote of reference 10, as this original emphasis (italics) no longer existed due to the journal’s formatting rules; the removal of this remark was overlooked during the last revision of the manuscript. The text passage now read as follows:

“As pointed out by Grimm et al. [56] and further elaborated on by Grimm and Eggel [75], the EU Directive itself explicitly states “may ultimately benefit human beings, animals or the environment” (Art. 38 p.2d [10]). Thus seemingly, and entirely contradictory to the concept of the HBA as a whole, allowing for situations where there is in fact no tangible benefit gained in the end, despite animals having been used. Instead, merely the prospect of achieving a beneficial outcome seems sufficient [75].”

Finally, in the original publication, **Alzmann (2016) (Ref. 117)** was not cited. The citation has now been inserted in **3. Results**, **3.11. Challenges and Opportunities Related to the Conception of Animal Research Ethics as a Field**, **3.11.1. Moral Pluralism in Animal Research Ethics** and should read as follows:

“While AECs are bound by the law, their task is morally far more demanding than simply “applying” existing law: “The standard of ethical decision-making for the majority of commission members was their intuition and personal conviction: They most frequently proceeded according to their intuition/their personal moral feeling […].” ([117], p. 71; own translation).”

With this correction, the order of some references has been adjusted accordingly. The authors state that the scientific conclusions are unaffected. This correction was approved by the Academic Editor. The original publication has also been updated.

**References**

29. WMA. Declaration of Helsinki. Ethical Principles for Medical Research Involving Human Participants. In Proceedings of the 75th WMA General Assembly, Helsinki, Finland, 16–19 October 2024; Available online: https://www.wma.net/policies-post/wma-declaration-of-helsinki/ (accessed on 30 September 2024).
46. Home Office (UK). Guidance on the Operation of the Animals (Scientific Procedures) Act 1986. Updated 2023. Available online: https://assets.publishing.service.gov.uk/media/66e0233ab1a8879e443282a0/Guidance_on_the_operation_of_ASPA_-_December_2023.pdf (accessed on 30 September 2024).
48. National Research Council (US) Committee for the Update of the Guide for the Care and Use of Laboratory Animals. *Guide for the Care and Use of Laboratory Animals*, 8th ed.; The National Academies Collection: Reports Funded by National Institutes of Health; National Academies Press: Washington, DC, USA, 2011. Available online: http://www.ncbi.nlm.nih.gov/books/NBK54050/ (accessed on 30 September 2024).
56. Grimm, H.; Eggel, M.; Deplazes-Zemp, A.; Biller-Andorno, N. The Road to Hell Is Paved with Good Intentions: Why Harm-benefit Analysis and Its Emphasis on Practical Benefit Jeopardizes the Credibility of Research. *Animals* **2017**, *7*, 70. https://doi.org/10.3390/ani7090070.
117. Alzmann, N.G. Zur Beurteilung der ethischen Vertretbarkeit von Tierversuchen. Ph.D. Thesis, Eberhard Karls Universität Tübingen, Tübingen, Germany, 2016. ISBN 978-3-7720-85557-4.
